# Insights into infancy weight gain patterns for term small-for-gestational-age babies

**DOI:** 10.1186/s12937-018-0397-z

**Published:** 2018-10-29

**Authors:** Huiqing Shi, Xiaodong Yang, Dan Wu, Xiulian Wang, Tingting Li, Honghua Liu, Chong Guo, Jian Wang, Xiangying Hu, Guangjun Yu, Jinjin Chen

**Affiliations:** 1 Shanghai Children’s Hospital, Shanghai Jiao Tong University, Luding Road 355, Putuo district, Shanghai, 200062 China; 2 Dongying People’s Hospital, Dongcheng South First Road 317, Dongying, 257091 China; 3 Maternal and Children Hospital, Daoshan Road 18, Gulou District, Fuzhou, 350001 China; 4 Jing’an District Maternal and Child Healthcare Center of Shanghai, 1297 Kangding Road, Shanghai, 200072 China

## Abstract

**Background:**

Too fast or slow weight gain in infancy is bad for health in later life. In this study, we aim to investigate the optimal weight gain pattern during the first 2 y of life for term small-for-gestational-age (SGA) infants.

**Method:**

We employed data from a longitudinal, community-based cohort study on the growth and development of SGAs collected between 2004 and 2010 in Shanghai, China.

Latent class growth analysis (LCGA) was applied to identify weight gain patterns among 3004 SGAs. BMI curves for each latent class from 1 mo to 5 y were produced through mixed-effects regression analysis. Multivariable regression was performed to examine the association between various classes and adverse outcomes (overweight/obesity/ malnutrition) during 2–5 y.

**Result:**

Five weight gain patterns aged 0–2 y of 3004 term SGAs were identified and labeled as follows--class 1: excessively rapid catch-up growth (10.7%); class 2: rapid catch-up growth (19.7%); class 3: appropriate catch-up growth (55.7%); class 4: slow catch-up growth (10.2%); class 5: almost no catch-up growth (3.7%). A decreasing age at adiposity rebound (AR) and an increasing BMI value were observed from class 5 to 1. Class 1 and 2 showed an early appearance of AR (< 4 y). SGAs in class 1 and 2 had a higher BMI in 2–5 y of life. After adjustment for potential confounding variables, class 1 and 2 were found to have an increased risk of being overweight/ obese. At the same time, we found the risk of malnutrition was especially prominent among SGAs in classes 4 and 5.

**Conclusion:**

Our results suggest that for term SGA infants, catch-up growth that crossing two centile levels, that is, from < 10th to the interval between 25th and 50th (ΔWAZ> 1.28) in the first several months, along with on track growth and maintenance at a median level by age 2 may be the optimal catch-up growth trajectory, minimizing risk of childhood adverse health outcomes.

## Background

Small-for-gestational-age (SGA) refers to newborns with birth weight (BW) below the 10th percentile of gender- and gestational age (GA)-specific reference (INTER- GROWTH-21st Project [1]). Most term SGA babies showed significantly rapid weight gain or catch-up growth (CUG) compensating for intrauterine restraint within the first two years of life [2]. Nevertheless, overweight and premature appearance of adiposity rebound (AR), which was reported to be a predictive marker of obesity and other metabolic syndromes in adulthood [3–5], can be observed in early childhood of SGA as well [6]. Growing evidences have suggested an increased long-term risk of excessive adiposity and the accompanying comorbidities across the life among infants who have been found to be with intrauterine growth restriction followed by rapid weight gain in infancy [7–9].

On the other hand, poor growth has also been associated with a variety of adverse health outcomes in later life [10–12]. Rapid weight gain has been shown to be strongly predictive of differences in stature [13]. Therefore, interventions for preventing slow growth and methods for promoting recovery from suboptimal growth have constantly been highly considered to be clinical priorities [14]. Both the WHO and CDC depict parallel growth charts for normal infants during the first two years of life, but babies who are in need of CUG ought to attain a higher weight gain rate during the same period [15].

Of equal importance to clinical intervention is appropriate growth monitoring, especially considering that excessive catch-up growth could lead to adverse outcomes in later life [16]. Pediatricians have been an indispensable part of the obesity prevention effort [17]. Yet, most only monitor infancy catch-up weight intensively when development is arrested, generally advising increases in energy intake for infants born with low weight [18]. Recommendations available concerning appropriate growth pattern for infants mostly do not account for the long-term health outcomes of these increased energy intakes. Current recommendations with respect to the time for starting overweight/obesity screening in childhood have generally not included infants: The U.S. Preventive Services Task Force (USPSTF), for example, recommends a screening window by the age of 6 y [19] and the WHO Experts Committee recommends screening by 2 y of age [20]. There is limited study available about the optimal growth trajectory for SGA catch-up growth, a need this work tries to address.

Through a retrospective longitudinal cohort study enriched with term SGA births, we sought to 1) identify common weight growth patterns during the first 2 y of life for term SGA young population; 2) investigate BMI growth patterns for SGA children in each trajectory class from 1 mo to 5 y of age and examine illustrative essential node (BMI value and age at AR) to assess the possibility of forward metabolic disorder; 3) test the association between the potential CUG trends and adverse outcomes in later life, revealing the pattern that has the lowest risk of obesity/overweight or malnutrition by preschool age.

## Methodology

### Study design

We employed data from a longitudinal, community-based cohort study on child growth of 32,307 SGA infants born between August, 2004 and July, 2010 in Shanghai, China. Anonymous GA, BW, gender and residence information were collected at birth and child anthropometric measurements were retrospectively extracted from health records of the Shanghai Center Disease Control Network. BWs were measured in birth hospitals or maternal healthcare centers and obtained according to maternal self-report during the infants’ first visit to the child health care center. Anthropometric measurements of weight, length/height were serially collected during the first 5 y through routine physical examination using standard anthropometric methods.

### Subjects

**Fig. 1:**
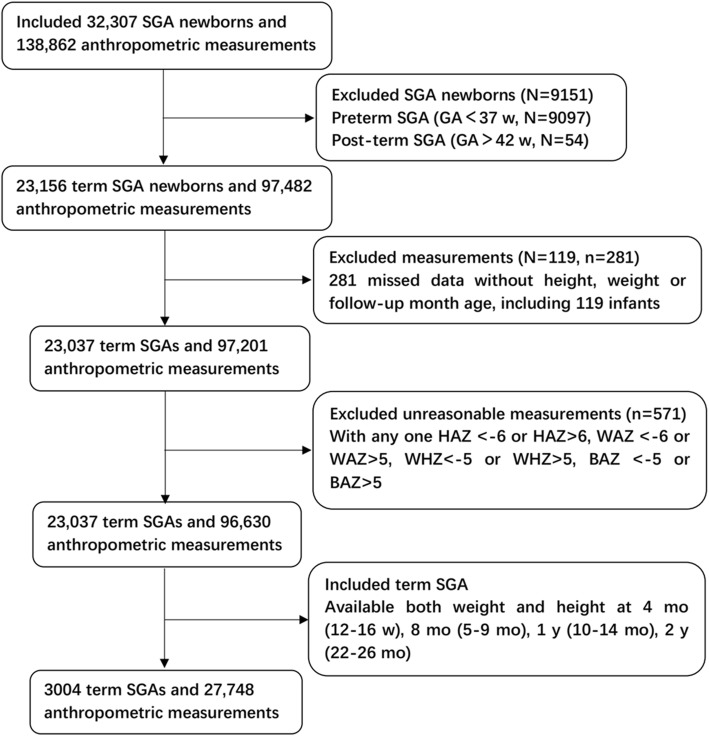
Flow chart for the study

In this study, newborns with BW below the 10th percentile for each corresponding GA were defined as SGA [1]. GA was assessed based on the last menstrual period and confirmed by early ultrasound pregnancy prior to 20 wk. of gestation. Among 32,307 SGAs, preterm (GA < 37 weeks, *N* = 9097) and post-term (GA > 42 weeks, *N* = 54) births were excluded from analysis because WHO growth trajectories are inapplicable to these infants [21]. Individual measurements with unreasonable data such as height-for-age Z-score (HAZ) < − 6 or HAZ > 6, weight-for-age Z-score (WAZ) < − 6 or WAZ > 5, weight-for-length Z-score (WHZ) < − 5 or WHZ > 5, BMI-for-age Z-score (BAZ) < − 5 or BAZ > 5 were excluded (*N* = 571) in the case of possible data-entry error [21]. Only data from infants with anthropometric measurements for both weight and height at each of following age points were used in this analysis: 4 mo (12–16 wk), 8 mo (5–9 mo), 1 y (10–14 mo), and 2 y (22–26 mo). This was done to obtain a more robust growth curve assessment at similar measurement dataset with comparable time points of the identified to generate weight-trajectory patterns. Of these data, only those with at least one follow-up evaluation during the period of 2–5 y were used. In total, 3004 SGAs were included in the final analysis (Fig. 1).

### Growth trajectory and profiling

Groupings of SGA growth patterns were identified according to the weight during the first 2 y of life. To find subgroups of children who shared similar linear growth profiles, we used a latent class growth analysis (LCGA), a technique employed to categorize individuals into distinct groups [22]. We used the 2006 WHO growth charts as a reference to calculate gender- and age-specific weight distributions prior to 2 y of age [23]. The weight-for-age Z-score (WAZ) in each time point was used for LCGA modeling. The WAZs was classified into 5 groups (<− 1.28, − 1.28 to (− 0.67), − 0.67 to 0.67, 0.67 to 1.28 and > 1.28, parallel with 10th, 25th, 75th and 90th percentile). The optimum number of growth profiles (latent classes) was selected on the basis of Bayesian information criterion (BIC) in order to possess the maximum likelihood of distribution into a specific latent class. Smaller value implied a better fit.

Deviations from normal physical growth were expressed as the prevalence of malnutrition such as children with Z-score < − 2 for height, weight, weight for height were defined as stunting, underweight, wasting, respectively. For the overnourished, overweight implied BAZ > 1 but < 2, while BAZ ≥ 2 was defined as obesity. Weight gain velocity between two target time points was indicated by different ΔWAZ degrees: first (ΔWAZ <− 0.67), second (− 0.67 ≤ ΔWAZ ≤0.67), third (ΔWAZ > 0.67 to 1.28), and fourth (ΔWAZ > 1.28). These categories are parallel to “crossing down one or more”, “no crossing”, “crossing up one” and “crossing up two or more” of the main weight centile on the WHO growth chart, respectively.

### Statistical analysis

Using LME4 in R, mixed-effects regression model was conducted to establish the 1 mo to 5 y BMI profiles for all five weight growth classes. All 3004 infants in our sample were eligible for growth modeling, because each had a minimum of three serial measurements, as described above. Age and BMI at AR were expressed as Amin and BMImin, respectively, and generated for each class to illustrate the group influence on BMI growth patterns. In addition, with the intention of identifying the independent effects of various growth patterns on adverse growth outcome (malnutrition /overweight /obesity), multiple linear regressions were adopted with adjustment for the following characteristics: gender, BW (2.5Kg ≤ BW < 3.0Kg, BW < 2.5Kg), GA (late term 40 ≤ GA<42 wk., early term 37 ≤ GA<40 wk.) [24], residence (urban, rural).

## Results

Table 1 shows the difference between SGAs included and excluded from our study. Most demographic characteristics were not significantly different among groups. By LCGA, the BIC values were 27,258.75,25,684.60,25,387.26,25,327.85, and 25,330.18 when the population was divided into 2–6 categories, of which five were optimal grouping number for the minimum BIC.

**Table 1:** Comparison of included and excluded term SGA of the study

	Term SGA included	Term SGA excluded	*P*-value	Total
Number of children [n (%)]	3004(12.9)	20,206(87.1)	–	23,210 (100.0)
Gender [n (%)]				
Male	1188(39.5)	8343(41.3)	0.07	9531(41.1)
Female	1816(60.5)	11,863(58.7)		13,679(58.9)
Gestational age (GA, wk.)	39.02(1.1)	39.13(1.1)	0.02	39.12(1.1)
GA categories [n (%)]				
Late term (40 wk. ≤ GA<42 wk.)	929(30.9)	5914(29.3)	0.09	6843(29.5)
Early term (37 wk. ≤ GA<40 wk.)	2075(69.1)	14,292(70.7)		16,367(70.5)
Birthweight (BW, Kg)	2.54(0.2)	2.51(0.2)	0.06	2.52(0.2)
BW (Z-score)	−1.70(0.5)	− 1.78(0.6)	< 0.01	− 1.77(0.5)
BW categories [n (%)]				
2.5Kg ≤ BW<3.0 Kg	2156(71.8)	14,233(70.4)	0.13	16,389(70.6)
BW<2.5Kg	848(28.2)	5973(29.6)		6821(29.4)
Residence [n (%)]				
Urban	486(16.2)	3425(16.9)	0.29	3911(16.8)
Rural	2518(83.8)	16,781(83.1)		19,299(83.2)

Continuous variables (gestational age, birthweight, BW (Z-score)) are shown in mean (SD)

**Fig. 2:**
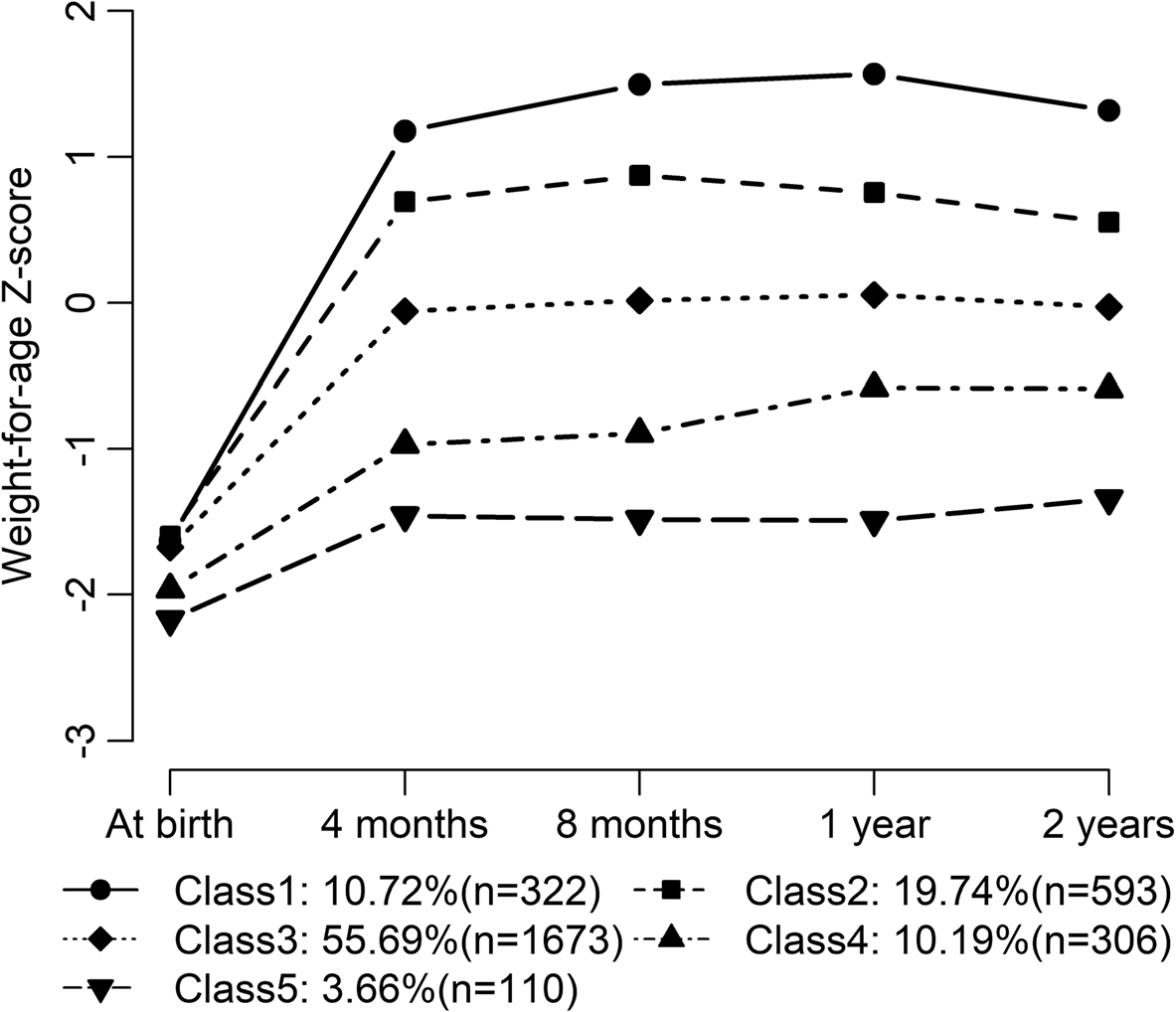
Trajectories of weight gain grouping classes in term SGA obtained by LCGA

Figure 2 presents the trajectories of different classes modeled via LCGA. The identified weight gain trajectory class 1, termed “excessively rapid catch-up growth”, accounted for 10.7% of the infants in our sample. This trajectory class possessed the characteristic of accelerating weight gain in the first 4 mo of life and persistence beyond risk level (WAZ ≥1) within the initial 2 y of life (WAZ = − 1.6, 1.2, 1.3 at birth, 4 mo and 2y, respectively). The weight gain class 2, named “rapid catch-up growth”, accounted for 19.7% of the subjects. During the first four months, these infants experienced excessively rapid growth but not as fast as class 1 and remained in the range between standard and risk level (0 ≤ WAZ ≤1) while under 2 y of age (WAZ = − 1.6, 0.7, 0.6 at birth, 4 mo and 2y, respectively). Trajectory class 3, termed “appropriate catch-up growth”, occupied over half of the infants (55.7%), also crossed centile rapidly in the four months while maintaining around median growth level thereafter (WAZ = − 1.7, − 0.1, 0.0 at birth, 4 mo and 2y, respectively). Trajectory class 4, termed “slow catch-up growth”, comprised 10.2% of the sample, featuring rapid growth at first and then leveling off modestly in the range between normal and lower limit (− 1 ≤ WAZ ≤ 0) afterwards (WAZ = − 2.0, − 1.0, − 0.6 at birth, 4 mo and 2 y, respectively). The representation of infants in the last trajectory class was 3.7%. This group exhibited “almost no catch growth” although there was rapid weight gain during the first four months. This gain did not sufficiently escalated above underweight risk levels (approximately − 2 to − 1 unit off median value), indicating suboptimal weight gain in accordance with WHO children growth standards (WAZ = − 2.2, − 1.5, − 1.3 at birth, 4 mo and 2 y, respectively).

Differences between the classes were observed for some characteristics. Heterogeneity of characteristics was accounted for by adjusting for some potential confounding factors. We further performed several analyses to detect the weight gain of infants in the first 4 mo of life (Table 2). Mean weight gain Z-score decreased by 2.1 unit across class 1 to 5 (*P* < 0.01). Infants from class 1–3 had a higher percentage of fourth (ΔWAZ > 1.28) and third weight gain degree (ΔWAZ > 0.67 to 1.28) than the other two classes during the first 4 mo of life. Most infants in class 4 and 5 were concentrated on the second (ΔWAZ from − 0.67 to 0.67) or third weight gain interval (WAZ < − 0.67).

**Table 2:** The baseline characteristics of the SGA by weight gain class

	Class 1 Excessively rapid catch-up growth	Class 2 Rapid catch-up growth	Class 3 Appropriate catch-up growth	Class 4 Slow catch-up growth	Class 5 Almost no catch growth	P for trend
Number of children [n (%)]	322(10.7)	593(19.7)	1673(55.6)	306(10.1)	110(3.6)	–
Gender [n (%)]						
Male	117(36.3)	201(33.8)	659(39.3)	145(47.3)	66(60.0)	< 0.01
Female	205(63.6)	392(66.1)	1014(60.6)	161(52.6)	44(40.0)	
Gestational age (GA, wk.)	39.06(1.1)	39.13(1.0)	39.06(1.0)	38.70(1.1)	38.70(1.2)	< 0.01
GA categories [n (%)]						
Late term (40 wk. ≤ GA<42 wk.)	102(31.6)	159(26.8)	490(29.2)	126(41.1)	52(47.2)	< 0.01
Early term (37 wk. ≤ GA<40 wk.)	220(68.3)	434(73.1)	1183 (70.7)	180(58.8)	58(52.7)	< 0.01
Birthweight (BW, Kg)	2.57(0.1)	2.58(0.1)	2.55(0.1)	2.45(0.2)	2.37(0.2)	< 0.01
BW (Z-score)	−1.63(0.4)	−1.60(0.4)	−1.68(0.4)	−1.96(0.6)	−2.17(0.6)	< 0.01
BW categories [n (%)]						
2.5Kg ≤ BW<3.0Kg	248 (77.0)	466(78.5)	1255(73.8)	165(53.9)	42(38.1)	< 0.01
BW<2.5Kg	74(22.9)	127(21.4)	438(26.1)	141(46.0)	68(61.8)	
Residence [n (%)]						
Urban	65 (20.1)	100(16.8)	249 (14.8)	56 (18.3)	16(14.5)	0.12
Rural	257(79.8)	493(83.1)	1424(85.1)	250(81.7)	94(85.4)	
ΔWeight-for-age Z-score degrees between 4 mo and birth[n (%)]	2.81(0.6)	2.29(0.6)	1.62(0.5)	0.99(0.6)	0.71(0.6)	< 0.01
First (ΔWAZ <−0.67)	0(0.0)	0(0.0)	1(0.0)	2(0.65)	1(0.91)	
Second (−0.67 < ΔWAZ< 0.67)	0(0.0)	4(0.6)	75(4.4)	99(32.35)	52(47.27)	
Third(0.67 < ΔWAZ<1.28)	6(1.8)	27(4.5)	403(24.0)	116(37.91)	40(36.36)	
Fourth (ΔWAZ> 1.28)	316(98.1)	56(94.7)	1194(71.3)	89(29.08)	17(15.45)	

Chi-square (categorical variables) or ANOVA (continuous variables) for linear trend, including all children (*n* = 3004); Data are expressed as mean ± SDS or No. (%).The Z-scores were calculated relative to age- and gender-specific WHO children growth standards. Continuous variables (gestational age, birthweight, BW (Z-score)) are shown in mean (SD)

**Fig. 3:**
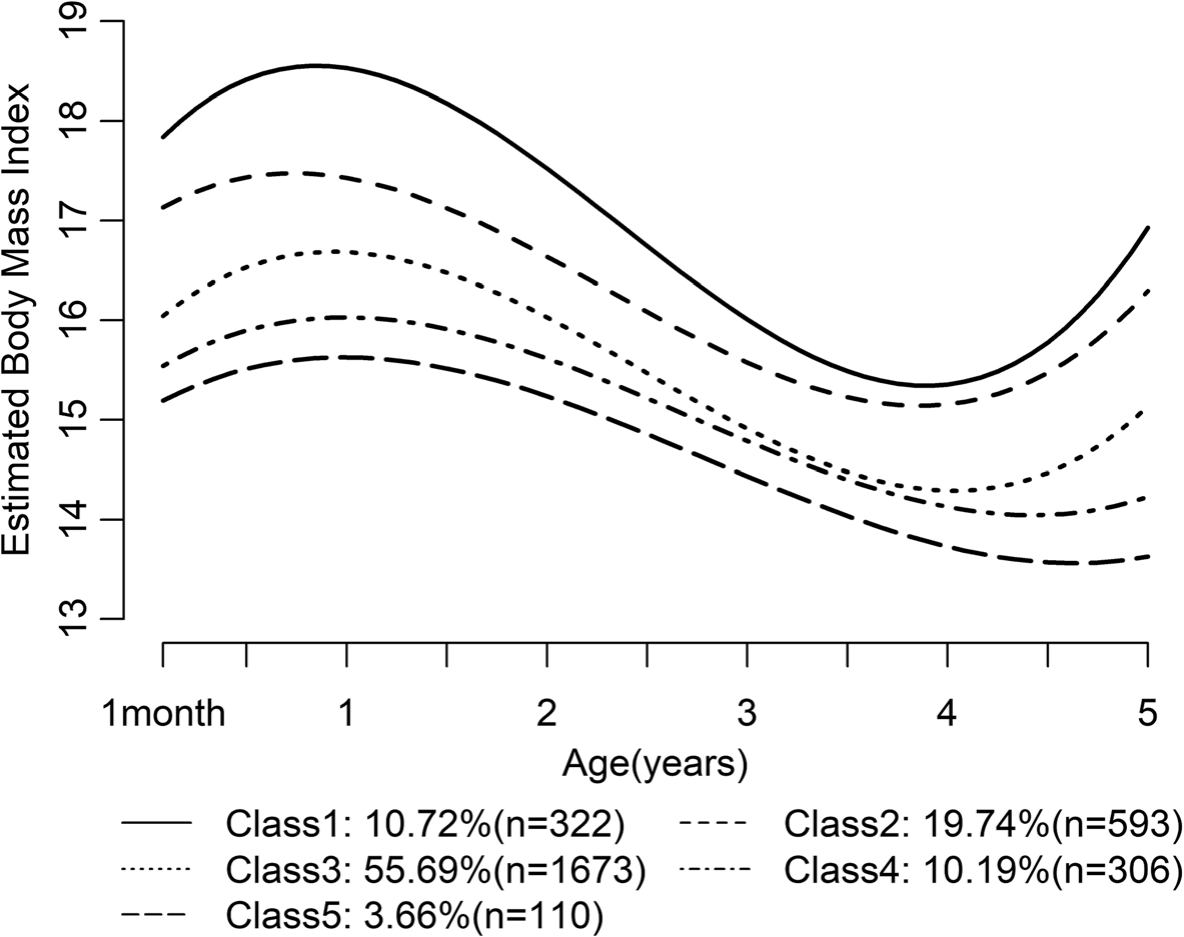
BMI growth trajectories age 1 mo to 5 y for each weight gain class

By using mixed-effects models, distance curves of BMI for each class were generated (Fig. 3). BMI commonly reaches the summit over the first year of life and descends afterward, to the lowest point around 5~ 7 y of age [25]. In this study, different classes showed different appearances of AR before 5 y of age (47, 46, 48, 53, 56 mo across class 1 to 5, respectively). Conversely, an increasing BMI was observed from class 5 to 1(13.6, 14.4, 14.3, 15.1, 15.3).

**Fig. 4:**
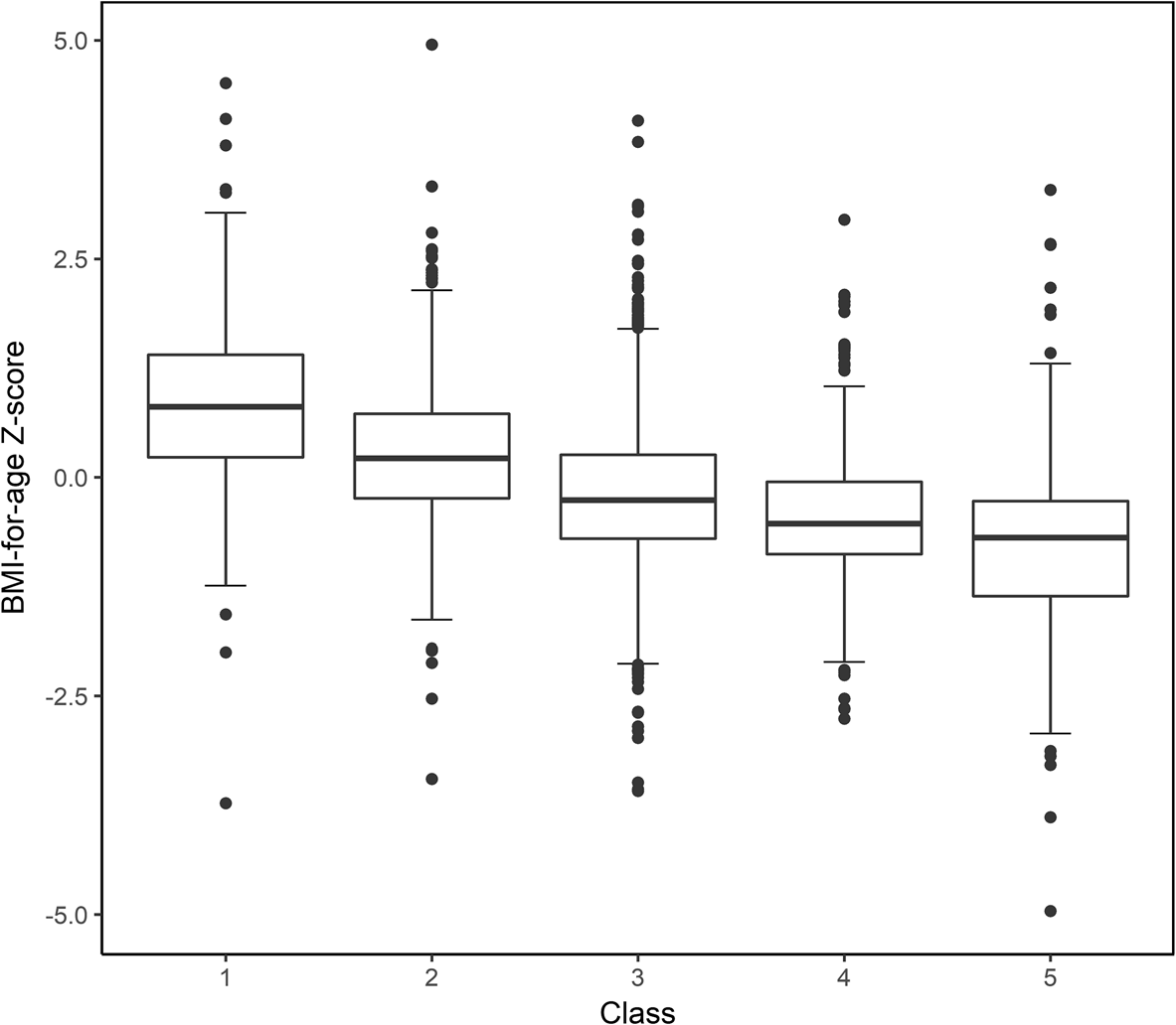
BMI Z-score when aged 2–5 y stratified by weight gain classes in the first 2 y

Weight gain classes in the first 2 y of life were associated with BMI z-score (Fig. 4) and rates of overweight/obesity (Table 3) during age 2–5 y. Table 3 shows when comparing with the class 3 (appropriate catch-up growth), SGAs in class 1 and 2 have a higher BMI age- and gender-specific Z-score during 2–5 y. The corresponding multivariate β value (95%CI) were 1.1 (1.0 to 1.2) for class 1 and 0.4 (0.4 to 0.5) for class 2, respectively, after adjustment for potential confounders. Extremely rapid weight gain (class 1) and rapid weight gain (class 2) in infancy increased the risk of overweight/obesity by 11 times (OR = 11.6; 95CI% from 8.8 to15.3) and 2 times (OR = 2.3; 95CI% from 1.8 to 3.0), respectively. An increased risk of malnutrition appeared to be particularly prominent among SGAs who were in class 4 and 5. After adjusting for other factors, the corresponding ORs (95%CI) were 4.4 (2.7 to 7.1) and 21.2 (12.6 to 35.6).

**Table 3:** BMI z-score and risks of adverse growth outcomes at age 2–5 y in each weight gain class

Trajectories classes	BMI-for-age Z-score	Overweight/obesity		Malnutrition	
	β (95CI %)	*N* (%)	OR (95CI %)	*N* (%)	OR (95CI %)
Class 1 Excessively rapid catch-up growth					
Unadjusted	1.1(1.0,1.2)^§^	174 (54.0)	10.6(8.1,13.9)^§^	6(1.9)	0.7(0.3,1.6)
Adjusted	1.1(1.0,1.2)^§^		11.6(8.8,15.3)^§^		0.7(0.3,1.6)
Class 2 rapid catch-up growth					
Unadjusted	0.4(0.4,0.5)^§^	118 (19.9)	2.2(1.7,2.9)^§^	2(0.3)	0.1(0.0,0.5)^‡^
Adjusted	0.4(0.4,0.5)^§^		2.3(1.8,3.0)^§^		0.1(0.0,0.5)^‡^
Class 3 Appropriate catch-up growth (referent)	1.0	167 (10.0)	1.0	45(2.7)	1.0
Class 4 Slow catch-up growth					
Unadjusted	−0.3(−0.4,-0.2)^§^	16 (5.2)	0.5(0.3,0.8)^‡^	34(11.1)	4.5 (2.8,7.2)^§^
Adjusted	−0.3(− 0.4,-0.2)^§^		0.5(0.3,0.8)^‡^		4.4(2.7,7.1)^§^
Class 5 Almost no catch-up growth					
Unadjusted	−0.7(−0.8,-0.5)^§^	5 (4.5)	0.4(0.2,1.1)^§^	42(38.2)	22.3(13.8,36.3)^§^
Adjusted	−0.7(− 0.8,-0.5)^§^		0.4(0.2,1.0)^§^		21.2(12.6,35.6)^§^

Adjusted for gender, birthweight (2.5Kg ≤ BW<3.0Kg, BW<2.5Kg), gestational age (late term 40 ≤ GA<42 wk., early term 37 ≤ GA<40 wk.), residence (urban, rural)

*CI* confidence interval, *OR* odds ratio

^‡^*P*<0.01;^§^*P*<0.001

## Discussion

Small-for-gestational-age births affect approximately 1 in 10 newborns worldwide. Several studies have been performed to demonstrate that rapid postnatal weight gain early in life is related to obesity later [26–29]. However, we are not aware of any work suggesting optimal growth patterns in infancy for reducing the risk of SGA obesity. The present study establishes the optimal postnatal weight gain pattern in the first two years of life by employing data from a large cohort study. Our study suggests that for term SGA infants, catch-up growth crossing two centile levels, that is, from < 10th to the interval between 25th and 50th (ΔWAZ> 1.28) in the first several months and maintaining on track growth at median level by two years of age is the optimal catch-up growth trajectory that presents the lowest risk of adverse health outcomes. Moreover, both inadequate and excessive catch-up growth patterns were found to be associated with poor outcomes. Our study demonstrated a significant difference in weight gain patterns in the early period of life, implicating the importance of early monitoring for SGAs.

As illustrated by DOHaD, low birth weight or SGAs have been reported to be associated with obesity and non-communicable diseases (NCD) [30, 31]. The emergence of AR at a younger age has been recognized as a predictor for obesity, NCD, and metabolic syndrome [4–6, 32]. Therefore, we characterized the timing of AR appearance in the different growth trajectory classes we modeled. Other researchers have presented early AR as the emergence of ascending BMI at ages under four [33]. In our work, both BMI and age of AR were found to be correlated with growth trajectory class. Earlier ARs and higher BMIs were observed in class 1 and 2: 2 and 1 mo earlier and 1.06 and 0.86 units higher than SGA children in class 3 (AR at 48 mo, BMI 14.28). The typical AR is the lowest BMI followed by an increased BMI after the rebound. Subsequent to the nadir in BMI growth trajectory, SGAs in each class showed accelerated BMI growth. The slope for this growth increased across classes (from class 5 to 1), which implicates a faster BMI growth for SGA children in school age. According to reports in previous literature, the BMI of SGA children with no infancy catch-up growth was low at 4 y [34], as depicted in class 5, confronting the challenge of malnourishment.

We also found that weight gain in the first two years was related to BMI-for-age Z-score, overweight/obesity, as well as malnutrition at preschool age in a dose-dependent pattern over all trajectories classes, even after adjustment for potential confounders. Overweight/obesity at preschool age was less prevalent in SGA class 4 and 5 than those (class 1, 2, 3) with faster weight gain growth in the same period. Excessive growth in early life appeared to be linked with less incidence of malnutrition at preschool age compared to class 4 and 5. However, it is essential to identify an optimal growth pattern to obtain balance between the requirement for ensuring sufficient intake versus the need to prevent childhood obesity and other associated health risks.

Our findings can be of real significance in clinical practice. Previous studies have reported that most SGA infants exhibit catch-up weight during early childhood [35, 36]. Sufficient infancy weight gain in SGAs appears to help prevent poor growth outcomes and may benefit neurodevelopment [25, 37]. Nevertheless, in spite of catch-up growth being beneficial, this study demonstrates that exceedingly fast weight gain in the first several months and a high weight later in infancy are both risk factors for overweight/obesity, necessitating appropriate weight gain control with cooperation between health workers and caregivers [38].

One of the limitations in this study is that the growth of a child is influenced by feeding status, nevertheless, it was not confirmed whether all SGAs consumed similar calories complying with feeding guideline of WHO. Secondly, the genetic potential (presented as the parental heights and weights) was unknown. The third is the lack of information about fetal life, such as maternal malnutrition, placenta origin, and genetic or chromosomal condition. Thus we were unable to detect the fetal origin of growth restrictions. It is quite likely the growth trajectories we obtained will vary considerably depending on different fetal growth histories. A fourth limitation is the limited information about health outcomes. In this study, we only possessed data about overweight/obesity and malnutrition. There are some other health indicators and biomarkers such as leptin and adiponectin that we were unable to assess, limiting the range of outcomes we could analyze. We also had a small number of babies with malnutrition, the result could be uncertain with wide confidence intervals. Despite the limitation, it is a large sample of community-based study covering complete ranges of age till 5 y. Furthermore, we conducted longitudinal analysis and evaluated growth by employing an effective and easy-to-fill screening method.

## Conclusion

In conclusion, suboptimal infant growth patterns of term SGAs are associated with adverse health outcomes during 2 to 5 years of age. Catch-up growth in SGA children is of great significance to erase the deficit at birth but should not extend to overgrowth or misbalanced growth. Monitoring and ensuring optimal catch-up growth starting from birth could be the first step towards prevention of childhood adverse outcomes.

## Abbreviations

AR: Adiposity rebound
BAZ: BMI-for-age Z-score
BMI: Body mass index
BW: Birth weight
CI: Confidence intervals
CUG: Catch-up growth
GA: Gestational age
HAZ: Height-for-age Z-score
LCGA: Latent Class Growth Analysis
OR: Odds ratios
SGA: Small-for-gestational-age
WAZ: Weight-for-age Z-score
WHZ: Weight-for-height Z-score

## References

[1] Villar J (2015). Postnatal growth standards for preterm infants: the preterm postnatal follow-up study of the INTERGROWTH-21(st) project. Lancet Glob Heal.

[2] Karlberg J, Albertsson-Wikland K (1995). Growth in full-term small-for-gestational- age infants: from birth to final height. Pediatr Res.

[3] Marakaki C, Karapanou O, Gryparis A (2017). Early adiposity rebound and premature Adrenarche. J Pediatr.

[4] Koyama S, Ichikawa G, Kojima M, Shimura N, Sairenchi T, Arisaka O (2014). Adiposity rebound and the development of metabolic syndrome. Pediatrics.

[5] Suchomlinov A, Tutkuviene J (2014). The relationship between birth weight, adiposity rebound and overweight at the age of 17 years (results of the Lithuanian longitudinal growth study, 1990 - 2008). Anthr Anz.

[6] Maeyama K (2016). Gestational age-dependency of height and body mass index trajectories during the first 3 years in Japanese small-for-gestational age children. Sci Rep.

[7] Singhal Atul (2017). Long-Term Adverse Effects of Early Growth Acceleration or Catch-Up Growth. Annals of Nutrition and Metabolism.

[8] Mortaz M, Fewtrell M, Cole T, Lucas A (2001). Birth weight, subsequent growth, and cholesterol metabolism inchildren 8-12 years old born preterm. Arch Dis Child.

[9] Cho WK, Suh BK (2016). Catch-up growth and catch-up fat in children born small for gestational age. Korean J Pediatr.

[10] Prado EL, Dewey KG (2014). Nutrition and brain development in early life. Nutr Rev.

[11] Gatyablonski G, Phillip M (2015). Nutritionally-induced catch-up growth. Nutrients.

[12] Raaijmakers A, Allegaert K (2016). Catch-up growth in former preterm neonates: no time to waste. Nutrients.

[13] Wright CM, Marie CK, Andrea S, Maria FV, Pearce MS, Adamson AJ (2012). To what extent do weight gain and eating avidity during infancy predict later adiposity?. Public Health Nutr.

[14] Norris S (2003). The relationship of rapid weight gain in infancy to obesity and skeletal maturity in childhood. Obesity.

[15] Oregon Pediatric Nutrition Practice Group. Nutrition Practice Care Guidelines for Preterm Infants in the Community. Available: http://www.oregon.gov/oha/.

[16] Okada T, Takahashi S, Nagano N, Yoshikawa K, Usukura Y, Hosono S (2015). Early postnatal alteration of body composition in preterm and small-for-gestational-age infants: implications of catch-up fat. Pediatr Res.

[17] Daniels SR, Hassink SG, Nutrition CO (2015). The role of the pediatrician in primary prevention of obesity. Pediatrics.

[18] Wang G, Sara J, Gong Y (2016). Weight gain in infancy and overweight or obesity in childhood across the gestational Spectrum: a prospective birth cohort study. Sci Rep.

[19] Barton M. Screening for obesity in children and adolescents: US preventive services task force recommendation statement. Pediatrics. 2015, 125(125):361–7. 10.1542/peds.2009-2037 20083515

[20] Bowdoin JJ (2008). Appendix: expert committee recommendations on the assessment, prevention, and treatment of child and adolescent overweight and obesity. Pediatrics.

[21] WHO AnthroPlus for personal computers Manual: Software for assessing growth of the world's children and adolescents. Geneva: WHO, 2009 (http://www.who.int/growthref/tools/en/ ).

[22] W (2006). M. G. R. S. group: WHO child growth standards based on length/height, weight and age. Acta Paediatr Suppl.

[23] Berlin KS, Williams NA, Parra GR (2014). An introduction to latent variable mixture modeling (part 1): overview and cross-sectional latent class and latent profile analyses. J Pediatr Psychol.

[24] A. C. O. Obstetriciansgynecologists, ACOG committee opinion no. 560 (2013). Medically indicated late-preterm and early-term deliveries. Obstet Gynecol.

[25] M. F. Rollandcachera, M. Deheeger, M. Maillot, and F. Bellisle: Early adiposity rebound: causes and consequences for obesity in children and adults. Int J Obes 2006, 30 Suppl 4(12):11. 10.1038/sj.ijo.0803514 17133230

[26] Longo S, Bollani L, Decembrino L (2013). Short-term and long-term sequelae in intrauterine growth retardation (IUGR). The journal of maternal-fetal & neonatal medicine : the official journal of the European Association of Perinatal Medicine, the Federation of Asia and Oceania Perinatal Societies, the International Society of Perinatal Obstetricians.

[27] Ong KK (2015). Postnatal growth in preterm infants and later health outcomes: a systematic review. Acta Paediatr.

[28] Woo Baidal JA, Locks LM, Cheng ER, Blake-Lamb TL, Perkins ME, Taveras EM (2016). Risk factors for childhood obesity in the first 1,000 days. A Systematic Review Am J Prev Med.

[29] Souza LG (2018). Predictors of overweight/obesity in a Brazilian cohort after 13 years of follow-up. Nutr J.

[30] Barker DJ, Osmond C (1986). Infant mortality, childhood nutrition, and ischaemic heart disease in England and wales. Lancet.

[31] Barker DJ, Osmond C, Forsén TJ, Kajantie E, Eriksson JG (2005). Trajectories of growth among children who have coronary events as adults. N Engl J Med.

[32] Wadsworth M, Butterworth S, Marmot M, Ecob R, Hardy R (2005). Early growth and type 2 diabetes: evidence from the 1946 British birth cohort. Diabetologia.

[33] Eriksson JG (2007). Epidemiology, genes and the environment: lessons learned from the Helsinki birth cohort study. J Intern Med.

[34] Taal HR, Vd Heijden AJ, Steegers EA, Hofman A, Jaddoe VW (2013). Small and large size for gestational age at birth, infant growth, and childhood overweight. Obesity.

[35] Leger J, Levy-Marchal C, Bloch J (1997). Reduced final height and indications for insulin resistance in 20 year olds born small for gestational age: regional cohort study. Bmj British Medical Journal.

[36] Hokkenkoelega ACS, Ridder MAJD, Lemmen RJ (1995). Children born small for gestational age: do they catch up[quest]. Pediatr Res.

[37] Varella MH, Moss WJ (2015). Early growth patterns are associated with intelligence quotient scores in children born small-for-gestational age. Early Hum Dev.

[38] Sunguya BF (2013). Effectiveness of nutrition training of health workers toward improving caregivers feeding practices for children aged six months to two years: a systematic review. Nutr Journal.

